# Effect of Corrosion-Induced Damage on Fatigue Behavior Degradation of ZCuAl_8_Mn_13_Fe_3_Ni_2_ Nickel–Aluminum Bronze Under Accelerated Conditions

**DOI:** 10.3390/ma18153551

**Published:** 2025-07-29

**Authors:** Ruonan Zhang, Junqi Wang, Pengyu Wei, Lian Wang, Chihui Huang, Zeyu Dai, Jinguang Zhang, Chaohe Chen, Xinyan Guo

**Affiliations:** 1 China Ship Scientific Research Center, Shanshui East Road No. 222, Binhu District, Wuxi 214082, China; zhangrn@cssrc.com.cn (R.Z.); wanglian@cssrc.com.cn (L.W.); daizy@cssrc.com.cn (Z.D.); 2 National Key Laboratory of Ship Structural Safety, Shanshui East Road No. 222, Binhu District, Wuxi 214082, China; 3 School of Civil Engineering and Transportation, South China University of Technology, Guangzhou 510640, China; 202420107071@mail.scut.edu.cn (J.W.); ctchhuang@mail.scut.edu.cn (C.H.); 202410181698@mail.scut.edu.cn (J.Z.); chenchaohe@scut.edu.cn (C.C.)

**Keywords:** ZCuAl_8_Mn_13_Fe_3_Ni_2_ nickel–aluminum bronze, corrosion accelerates, numerical simulation, fatigue behavior degradation

## Abstract

Corrosion fatigue damage significantly affects the long-term service of marine platforms such as propellers. Fatigue testing of pre-corrosion specimens is essential for understanding damage mechanisms and accurately predicting fatigue life. However, traditional seawater-based tests are time-consuming and yield inconsistent results, making them unsuitable for rapid evaluation of newly developed equipment. This study proposes an accelerated corrosion testing method for ZCuAl_8_Mn_13_Fe_3_Ni_2_ nickel–aluminum bronze, simulating the marine full immersion zone by increasing temperature, adding H_2_O_2_, reducing the solution pH, and preparing the special solution. Coupled with the fatigue test of pre-corrosion specimens, the corrosion damage characteristics and their influence on fatigue performance were analyzed. A numerical simulation method was developed to predict the fatigue life of pre-corrosion specimens, showing an average error of 13.82%. The S–N curves under different pre-corrosion cycles were also established. The research results show that using the test solution of 0.6 mol/L NaCl + 0.1 mol/L H_3_PO_4_-NaH_2_PO_4_ buffer solution + 1.0 mol/L H_2_O_2_ + 0.1 mL/500 mL concentrated hydrochloric acid for corrosion acceleration testing shows good corrosion acceleration. Moreover, the test methods ensure accuracy and reliability of the fatigue behavior evaluation of pre-corrosion specimens of the structure under actual service environments, offering a robust foundation for the material selection, corrosion resistance evaluation, and fatigue life prediction of marine structural components.

## 1. Introduction

Corrosion is an electrochemical process characterized by progressive degradation and material loss resulting from the interaction of materials with their surrounding environment. This phenomenon is particularly pronounced in metals, where it severely compromises structural integrity and reduces service life. In natural marine environments, the corrosion behavior of metals is influenced by a complex interplay of multiple factors, including dissolved oxygen concentration, salinity, temperature, and biological activity [1]. When designing tests to simulate seawater corrosion conditions, it is essential to meticulously control these factors, as they critically influence the corrosion kinetics and underlying mechanisms of material degradation. A comprehensive understanding of these factors is vital for developing effective corrosion mitigation strategies and enhancing the longevity of marine structures.

The ZCuAl_8_Mn_13_Fe_3_Ni_2_ nickel–aluminum bronze is extensively utilized in propeller dynamic positioning systems for deepwater oil and gas exploration equipment, such as semi-submersible drilling platforms, owing to its outstanding comprehensive properties, including superior corrosion resistance, excellent mechanical performance, and remarkable biofouling resistance. Deepwater semi-submersible platforms generally employ two positioning technologies: mooring positioning and dynamic positioning. Under normal circumstances, when operating in relatively shallow waters (less than 1500 m), independently controlled anchors are deployed for positioning. However, as water depth increases, the mooring system’s holding capacity diminishes, and anchor deployment becomes progressively more challenging. Consequently, dynamic positioning systems are typically employed, utilizing multiple azimuth thrusters capable of 360° rotation to continuously adjust the vessel’s position. The propellers face dual operational challenges. In shallow-water conditions, prolonged seawater immersion leads to micro-pitting corrosion on surfaces. This creates localized stress concentrations in blade structures. These stress concentrations reduce material lifespan and strength reserves. In deepwater environments, propeller blades endure high-amplitude alternating loads. These loads result from ocean currents superimposed with operational loads. As service time progresses, these harsh operating conditions and stringent performance requirements pose significant challenges to fatigue resistance. If the fatigue resistance does not meet the requirements, it will seriously threaten people’s property and life safety. Statistical data indicate that corrosion fatigue fracture constitutes the primary failure mode for nickel–aluminum bronze propellers, accounting for approximately three-quarters of all propeller failures [2,3,4,5]. To ensure operational safety, post-corrosion fatigue strength evaluation becomes imperative, making research on seawater corrosion’s impact on nickel–aluminum bronze material fatigue performance critically important. The actual service life of materials typically extends over several years. While natural seawater exposure testing can accurately simulate material-environment interactions, this method is characterized by excessively long test cycles (often requiring multi-year durations), coarse experimental data with limited precision, and poor reproducibility between parallel samples. In contrast, laboratory-based simulated testing can obtain material corrosion data within significantly shorter timeframes, proving highly valuable for evaluating corrosion resistance and supporting material selection decisions. The current detailed corrosion resistance specifications for marine propellers include (1) Salt Spray Test (ASTM B117): ≥1000 h without pitting corrosion; (2) Corrosion Rate (Seawater, 25 °C): <0.05 mm/year (per ASTM G31); (3) Cavitation-Erosion Resistance: Mass loss < 5 mg/cm^2^ (per ASTM G32, 10 h test); (4) Galvanic Corrosion Potential: −0.25 to −0.35 V (vs. Ag/AgCl).

Currently, several accelerated corrosion testing methods are widely employed to evaluate the corrosion resistance of metallic materials. These methods include [6]: (1) the salt spray test, which simulates marine or industrial environments with high chloride concentrations; (2) the moist heat test, which assesses material performance under high temperature and humidity conditions; (3) the cyclic immersion test, which alternates between wet and dry phases to simulate tidal or splash zone exposures; (4) the electrolytic accelerated corrosion test, which uses electrochemical techniques to accelerate the corrosion process; (5) the multi-factor cyclic composite test, which combines multiple environmental factors to simulate complex real-world conditions.

The moist heat test [6] simulates specific temperature and humidity conditions using a controlled environmental chamber to intensify the corrosion environment for metals. However, this method has limitations in accurately replicating the actual corrosion mechanisms observed in real-world scenarios. The cyclic immersion test [6] is performed by fully, partially, or periodically immersing metal specimens in a corrosive environment. This method is widely regarded as one of the most common accelerated corrosion testing techniques due to its ability to simulate realistic wet–dry cycling conditions [7]. The indoor accelerated corrosion test employing this method demonstrates a strong correlation with real-sea exposure tests, making it a viable alternative for simulating marine environmental conditions [8]. The electrolytic accelerated corrosion test [6] is an efficient technique for assessing the corrosion resistance of deposited layers on metal castings, with broad applications in material evaluation and quality control. The multi-factor cyclic composite test [6] integrates cyclic immersion tests, alternating between wet and dry phases, with periodic salt spray to simulate more complex environmental conditions. By formulating a simulated environmental acceleration spectrum [9], key parameters can be precisely controlled to enhance the correlation between indoor accelerated corrosion test results and actual field performance.

Current research on the corrosion of nickel–aluminum bronze primarily focuses on three key areas: (1) the corrosion behavior of nickel–aluminum bronze; (2) strategies to enhance its corrosion resistance; and (3) the impact of alloying element content on the corrosion resistance of nickel–aluminum bronze [10]. However, there is a notable lack of studies on indoor accelerated corrosion testing of nickel–aluminum bronze. Several researchers have performed accelerated corrosion tests on copper alloys [11]. The findings reveal that the sequence of corrosion product formation during the neutral salt spray test closely resembles that observed in real-world conditions.

Marine corrosion of metallic materials primarily manifests as pitting corrosion, galvanic corrosion, and crevice corrosion, among others [12]. Various numerical simulation methods [13] have been developed to study these corrosion mechanisms, including cellular automaton simulation, finite element simulation, and boundary element simulation. Galvanic corrosion, which occurs due to differences in the self-corrosion potentials of dissimilar metals in the same electrolyte, is typically simulated using the finite element method or the boundary element method. The thickness and concentration of the electrolyte liquid film significantly influence the current density of galvanic corrosion [14], with thicker liquid films generally resulting in higher current densities [15]. Pitting corrosion, a form of localized galvanic corrosion occurring on the same metal, is readily induced by chloride ions in the electrolyte. This phenomenon is frequently simulated using cellular automata [13]. While extensive research has been conducted by scholars worldwide on the morphological evolution of pitting corrosion sites [16,17], fewer studies have focused on their growth and development [13]. Similarly, crevice corrosion, another localized corrosion phenomenon, is often simulated using the finite element method [13]. The primary driver of crevice corrosion is the oxygen concentration difference, with lower dissolved oxygen levels inside the crevice and higher levels outside. Passive metallic materials, such as stainless steel, are particularly susceptible to crevice corrosion.

Numerous studies have demonstrated that pre-corrosion treatment significantly degrades the fatigue properties of metal specimens. However, the majority of these studies focus on steel, with limited research conducted on alloys such as nickel–aluminum bronze. Given its critical role in the construction of marine facilities, including ships, it is essential to study the fatigue properties of nickel–aluminum bronze after undergoing pre-corrosion treatment. Knysh [18] subjected low-alloy steel to salt spray pre-corrosion treatment, resulting in a 15% reduction in the fatigue performance of the specimens. Liang [19] applied salt spray pre-corrosion treatment to Q355NHD weathering steel and discovered a 36.5% decrease in fatigue strength at 2 × 10^6^ cycles. Zhang [20] treated Q345 steel with salt spray pre-corrosion and discovered that fatigue cracks initiated from pitting sites, leading to a 22.6% reduction in fatigue strength. Chen [21] conducted salt spray pre-corrosion treatment on HRB400E steel for varying durations, discovering a significant decline in the fatigue life of the specimens. Nikolaos [22] investigated the fatigue properties of the innovative Al-Cu-Li (2198) aluminum alloy after corrosion exposure and compared it with the Al-Cu (2024) alloy. They found that after more than 12 h of pre-corrosion treatment, the Al-Cu (2024) alloy lost nearly 30% of its initial ultimate tensile strength, whereas the Al-Cu-Li (2198) alloy lost only 11%. Sangshik [23] evaluated the impact of pre-corrosion on the fatigue life of Al–Zn–Mg–Cu alloys, attributing the reduction in fatigue life to clustered corrosion pits on the L–S surface induced by laboratory-EXCO exposure.

This study integrates numerical simulation with accelerated corrosion testing to develop a high-efficiency accelerated corrosion testing method. The results are validated through comparison with real-sea exposure tests, ensuring the accuracy and reliability of the method. Additionally, experimental investigations were conducted to evaluate the fatigue life and strain variations of nickel–aluminum bronze specimens subjected to different pre-corrosion durations.

## 2. Accelerated Corrosion Test and Methods for Nickel–Aluminum Bronze in Marine Environments

### 2.1. Electrochemical Properties of Nickel–Aluminum Bronze in Accelerated Corrosion Conditions

Polarization curves provide an intuitive characterization of electrochemical corrosion processes. By analyzing corrosion potential and corrosion current under accelerated corrosion conditions, the corrosion rate and its influencing factors can be effectively evaluated. To design accelerated corrosion conditions and obtain electrochemical parameters, electrochemical performance tests were conducted on ZCuAl_8_Mn_13_Fe_3_Ni_2_ nickel–aluminum bronze.

The material investigated in this study is ZCuAl_8_Mn_13_Fe_3_Ni_2_ nickel–aluminum bronze, hereafter referred to as nickel–aluminum bronze. The primary chemical composition and mass fractions of nickel–aluminum bronze are shown in Table 1.

Polarization curve testing of nickel–aluminum bronze was conducted in natural seawater, artificial seawater, and five specifically designed accelerated corrosion test conditions. The detailed accelerated corrosion testing conditions are shown in Table 2.

The natural seawater utilized in this study was sourced from the coastal region of Qingdao. In accordance with the standard GB/T 17848-1999 [25], the chemical composition of the artificial seawater is shown in Table 3.

**Figure 1:**
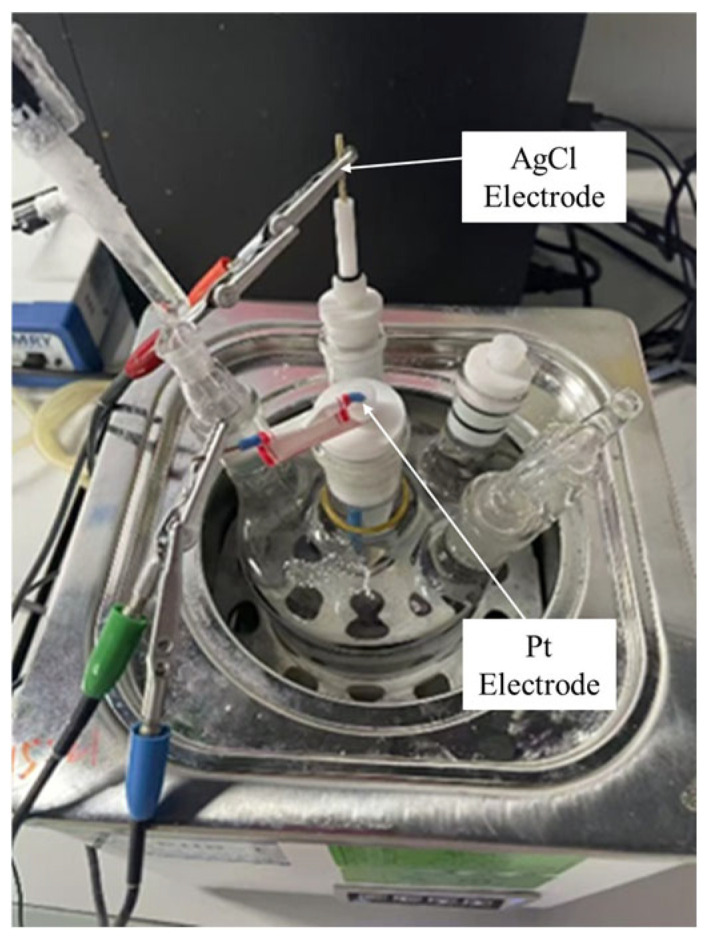
Electrochemical workstation.

Nickel–aluminum bronze rods with a diameter of 10 mm were machined as working electrodes. Prior to testing, one end of each rod was polished to a mirror-like finish. To ensure accurate exposure area during measurements, the polished surface was exposed to the test solution, while the remaining surfaces were sealed. The test solutions included natural seawater and multiple accelerated corrosion solutions. A three-electrode system was employed, consisting of an AgCl reference electrode, a Pt counter electrode, and the nickel–aluminum bronze working electrode, connected to a CS310M (JF69) electrochemical workstation. As shown in Figure 1.

The results of the electrochemical performance parameter analysis and polarization curves for the seven experimental groups are also shown in Table 2.

**Figure 2:**
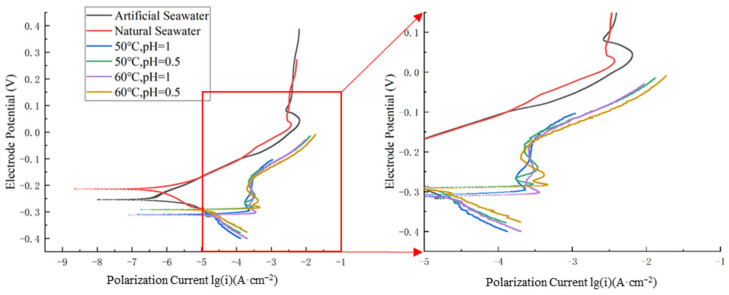
Polarization curves of nickel–aluminum bronze in different environments (excluding special solutions).

As shown in Figure 2, the polarization curves obtained in the artificial seawater condition closely match those in the natural seawater condition. Additionally, the self-corrosion current density and self-corrosion potential demonstrate similar values in both conditions.

As shown in Figure 2 and Table 2, higher temperatures and lower pH values significantly enhance the corrosion rate of nickel–aluminum bronze. At a constant temperature of 50 °C, decreasing the pH from 1 to 0.5 leads to an increase in the corrosion current density from 7.943 μA·cm^−2^ to 11.480 μA·cm^−2^, representing an increase of 3.537 μA·cm^−2^ (44.53% increase). This indicates that a reduction in pH accelerates the corrosion process.

**Figure 3:**
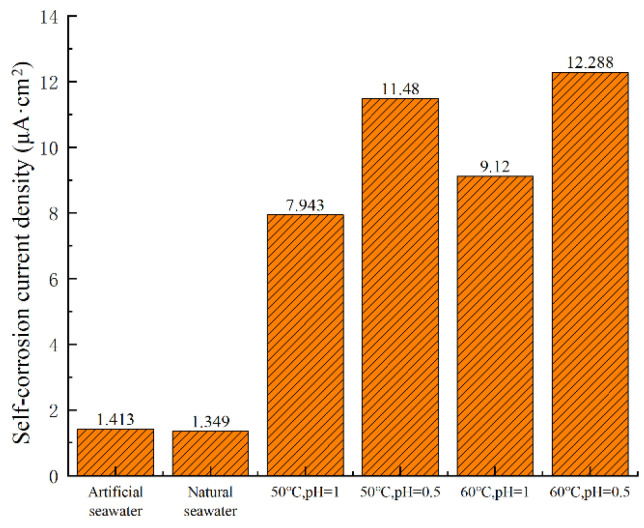
Corrosion current density under different conditions.

Furthermore, the data in Figure 2 and Table 2 show that at a constant pH of 1, increasing the temperature from 50 °C to 60 °C leads to a rise in the corrosion current density from 7.943 μA·cm^−2^ to 9.120 μA·cm^−2^, representing an increase of 1.177 μA·cm^−2^ (14.82% increase). Similarly, at a constant pH of 0.5, increasing the temperature from 50 °C to 60 °C leads to a rise in the corrosion current density from 11.480 μA·cm^−2^ to 12.288 μA·cm^−2^, representing an increase of 0.808 μA·cm^−2^ (10.17% increase). Compared with the 3.537 μA·cm^−2^ increase in corrosion current density caused by a pH reduction at 50 °C, it is evident that a decrease in pH has a more significant impact on accelerating the corrosion rate of nickel–aluminum bronze than a temperature increase. The corrosion current density diagram under different environments (except for special solutions) is shown in Figure 3.

**Figure 4:**
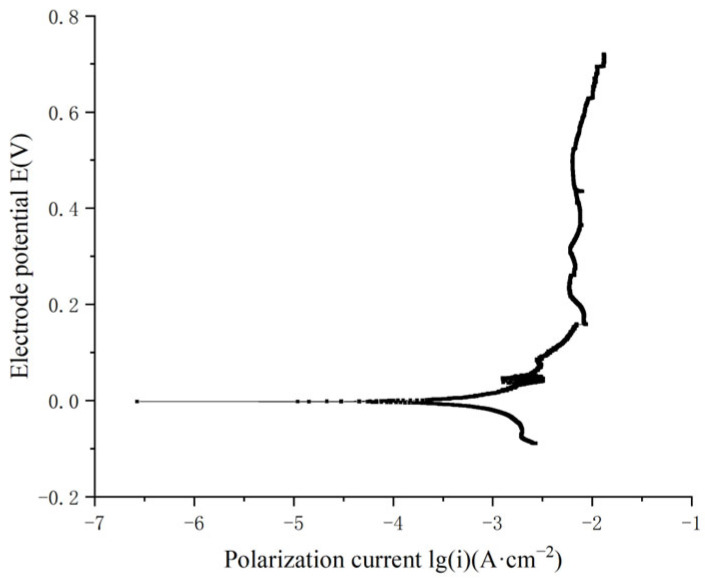
Polarization curve of nickel–aluminum bronze in the special corrosion solution.

To study the combined effects of corrosion and fatigue on the mechanical properties of materials, it is essential to develop an accelerated corrosion condition that significantly intensifies conditions compared with natural seawater. Current polarization curve tests reveal that increasing temperature alone places high demands on experimental apparatus, while reducing the pH of the solution alone has limited effectiveness. Based on relevant research [24], this study proposes a special corrosion condition comprising 0.6 mol/L NaCl, 0.1 mol/L H_3_PO_4_, 0.1 mol/L NaH_2_PO_4_ buffer solution, and 1 mol/L H_2_O_2_, with a hydrochloric acid volume fraction of 0.1 mL/500 mL. Polarization curve tests for nickel–aluminum bronze were performed in this special corrosion condition at a constant temperature of 25 °C, and the results shown in Figure 4.

From Table 2 and Figure 4, it is evident that the corrosion reaction of nickel–aluminum bronze is notably severe in the hydrogen peroxide solution. Based on Faraday’s law, the self-corrosion current density is directly proportional to the material’s corrosion rate, with a higher self-corrosion current density indicating a faster corrosion rate. In the accelerated corrosion test condition at 25 °C, the solution consisted of 0.6 mol/L NaCl, 0.1 mol/L H_3_PO_4_, 0.1 mol/L NaH_2_PO_4_ buffer solution, 1 mol/L H_2_O_2_, and 0.1 mL/500 mL hydrochloric acid. The self-corrosion current density of nickel–aluminum bronze reached 288.403 μA·cm^−2^, demonstrating a significant acceleration of corrosion.

### 2.2. Corrosion Testing in Accelerated Corrosion Conditions

Based on the accelerated solution established in the previous section, this section conducts accelerated corrosion testing to accurately determine the corrosion rate and assess the surface damage morphology.

#### 2.2.1. Corrosion Test

**Figure 5:**
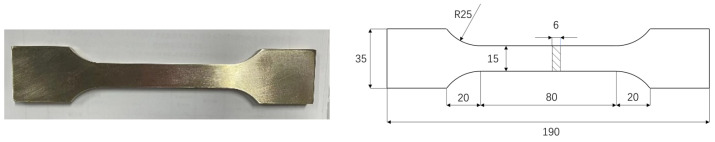
Test specimen of nickel–aluminum bronze.

Following the requirements of GB/T 6384 [26], the thickness of test specimens is set at 6 mm. Two different cases were considered for accelerated corrosion tests, with specific dimensions of the specimens shown in Figure 5. One is exposed to corrosion for 15 days and the other for 30 days. Conditions at 25 °C comprise a solution with the special corrosion solution.

**Figure 6:**
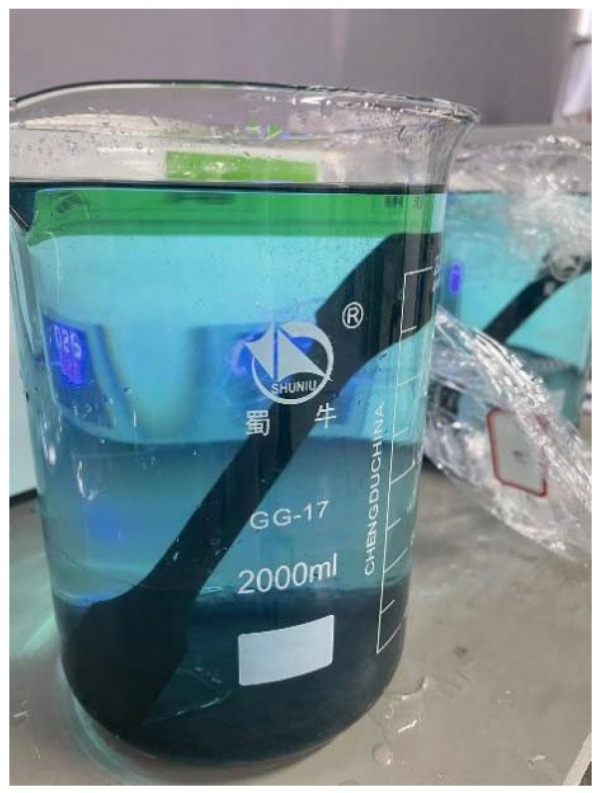
Corrosion test.

Before the corrosion test, the specimen’s surface was polished progressively until all oxidation residues and impurities were removed, resulting in a smooth surface. To ensure consistent surface conditions between control and pre-corroded specimens, all specimens (including corrosion-free controls) underwent identical polishing procedures. Each specimen was then weighed three times using an analytical balance with an accuracy of 0.0001 g, and the average of these three weights was taken as the weight of the specimen before the corrosion test. After preparing the accelerated corrosion solution, each of the test specimens was sealed in a beaker, as shown in Figure 6. To ensure the stability of the temperature during the test, the beaker was placed in a constant-temperature water bath maintained at 25 °C for the corrosion test. During the entire test process, the corrosion solution was replenished every 7.5 days to avoid significant variations in its chemical composition.

#### 2.2.2. Accelerated Corrosion Weight Loss Analysis

**Figure 7:**
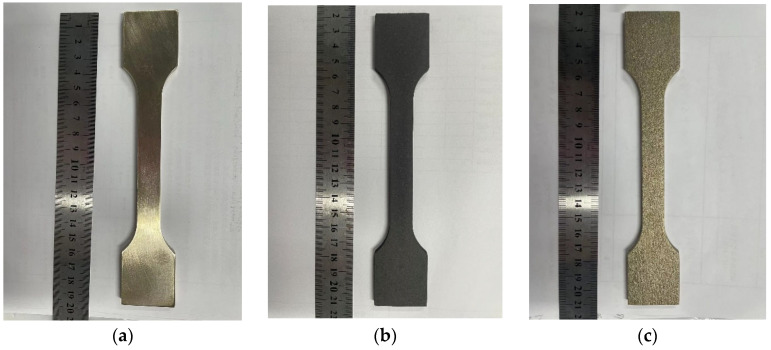
15d experimental group: nickel–aluminum bronze specimens before corrosion (**a**), after corrosion (**b**), and post-corrosion product removal (**c**).

**Figure 8:**
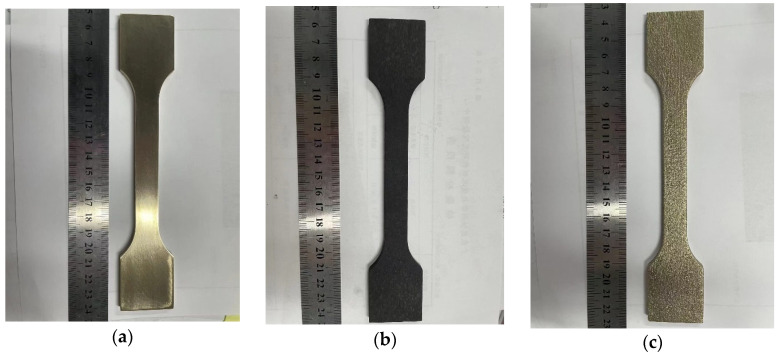
30d experimental group: nickel–aluminum bronze specimens before corrosion (**a**), after corrosion (**b**), and post-corrosion product removal (**c**).

The 15d and 30d corroded specimens are shown in Figure 7 and Figure 8, respectively.

The corrosion products were removed using a three-step cleaning process: (1) Chemical cleaning: specimens were immersed in 10% citric acid solution and ultrasonicated for 30 min (300 W, 50 °C); (2) Mechanical polishing: residual products were gently removed by transverse grinding with 2000# SiC sandpaper; (3) Rinsing with deionized water, dehydrating with anhydrous ethanol, and drying with cold air.

After removing the corrosion products from the surface of the specimens, their weight loss is measured. The corrosion rate of nickel–aluminum bronze is 2.3858 g/m^2^·h during the 15d test, with an estimated weight loss of 118.526 g after 0.5 years. In the 30d test, the corrosion rate of nickel–aluminum bronze is 2.4577 g/m^2^·h, and the estimated weight loss of the specimen after 0.5 years is 122.098 g.

According to relevant studies, the corrosion rates of copper–nickel alloy specimens in real-sea environments change very little within 0.5 years [27]. The real sea test results of nickel–aluminum bronze specimens showed that the average corrosion rate is 0.02 mm/a. Based on the corrosion specimen size, it is calculated that the weight loss of nickel–aluminum bronze is 1.0421 g after 0.5 years under natural seawater conditions. Compared with the corrosion rate of a real marine environment, the accelerated corrosion rate of the 15d corrosion test reached 113.23 times, and that of the 30d corrosion test reached 117.17 times. In the 15d accelerated corrosion test, the weight loss rate of nickel–aluminum bronze was 2.3858 g/m^2^·h, equivalent to 4.67 years of corrosion in natural seawater, based on the average corrosion rate of 0.0211 g/m^2^·h. Similarly, in the 30d accelerated corrosion test, the weight loss rate of nickel–aluminum bronze was 2.4577 g/m^2^·h, equivalent to 9.63 years of corrosion in natural seawater.

#### 2.2.3. Scanning of Surface Corrosion Morphology at Different Corrosion Stages

**Figure 9:**
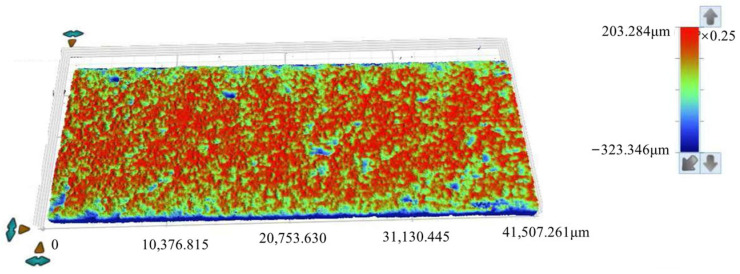
Topographical surface images acquired via white light scanning of specimens after 15 days of corrosion.

**Figure 10:**
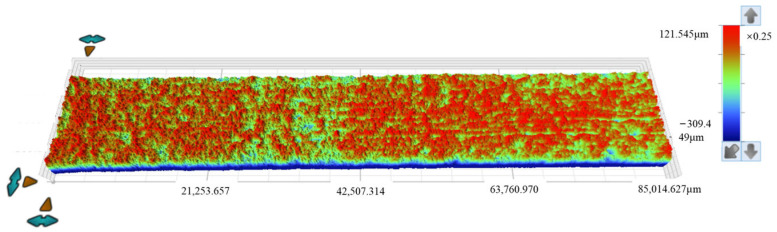
Topographical surface images acquired via white light scanning of specimens after 30 days of corrosion.

After removing the corrosion products, white light testing was performed on the specimens. The white light testing was conducted using a three-dimensional optical profiler, model Contour X-500, manufactured by Bruker corporation (Tempe, AZ, USA). Roughness analysis was conducted on the scanned images to determine the roughness parameters for different sections of the specimens. The surface morphology images are shown in Figure 9 and Figure 10, while the roughness parameters are shown in Table 4.

The zero-height reference plane was defined using Vision64 software (Bruker Contour X-500 three-dimensional optical profiler, manufactured by Bruker corporation, Tempe, Arizona, USA). A flat-edge region without significant corrosion pits was selected, and a plane was fitted to this region using the ‘least squares plane fitting’ algorithm. This fitted plane was set as the zero-height reference (z = 0), and the height data range represents relative heights from this plane.

The roughness parameters include the standard deviation of height distribution (*S*_q_), the average height difference (*S*_a_), the maximum height (*S*_p_), the minimum height (*S*_v_), the difference between height extremes (*S*_z_), the skewness of the height distribution (*S*_sk_), and the kurtosis of the height distribution (*S*_ku_). In Figure 9 and Figure 10, the *S*_sk_ value is negative, indicating that the height data is concentrated in the higher value region, while the *S*_ku_ value exceeds 3, suggesting the height distribution of the specimen surface is sharp and needle-like.

As shown in Table 4, for the 15d and 30d specimens, the average height difference (*S*_a_) increased from 16.897 µm (15d) to 29.03 µm (30d), suggesting progressive surface roughening and intensified corrosion over time. The kurtosis of the height distribution (*S*_ku_) decreased from 14.48 (15d) to 10.699 (30d), suggesting surface features became flatter with advancing corrosion. The skewness of the height distribution (*S*_sk_) decreased from −3.001 (15d) to −2.292 (30d), suggesting reduced asymmetry in height distribution and more uniform corrosion morphology. The maximum height (*S*_p_) decreased from 203.284 µm (15d) to 121.571 µm (30d), suggesting that attenuation of surface protrusions is likely associated with corrosive degradation. The difference between height extremes (*S*_z_) decreased from 526.630 µm (15d) to 431.395 µm (30d), suggesting an overall reduction in surface topography extremes. The standard deviation of height distribution (*S*_q_) increased from 34.628 µm (15d) to 47.556 µm (30d), suggesting greater variability in roughness and heightened surface complexity.

Parameter analysis suggests the following trends from prolonged corrosion exposure. First, the specimen’s surface roughness increases progressively. Second, overall surface height characteristics decrease. Third, surface asymmetry and variability become more pronounced. These changes collectively reflect two key points: corrosion is persistent, and it substantially impacts the evolution of surface morphology.

## 3. Fatigue Properties of Specimens After Accelerated Corrosion

To study the influence of accelerated corrosion conditions on the fatigue properties of nickel–aluminum bronze, tensile fatigue tests were conducted on specimens under three states: no corrosion, pre-corroded for 15 days, and pre-corroded for 30 days. The accelerated corrosion solution was the special solution used in Section 2.

### 3.1. Fatigue Test and Specimens

**Figure 11:**
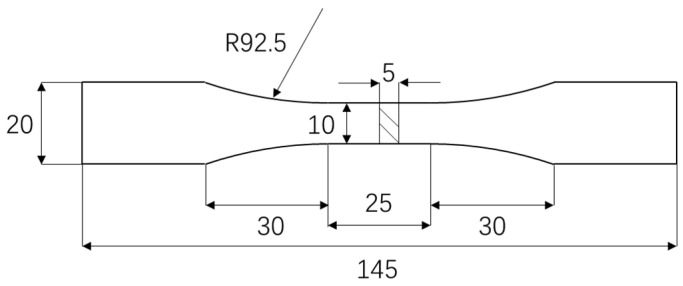
Fatigue specimen (unit: mm).

The test utilized ZCuAl_8_Mn_13_Fe_3_Ni_2_ nickel–aluminum bronze. The dimension of the fatigue test specimens is shown in Figure 11.

**Figure 12:**
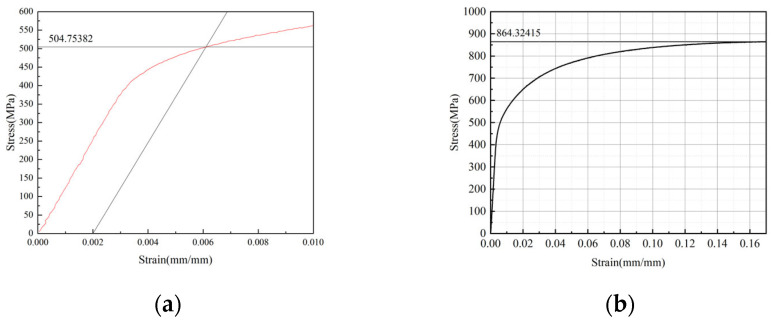
Stress–strain curve (**a**) the yield strength (at 0.2% residual deformation); (**b**) the tensile strength.

Tensile properties were measured on uncorroded virgin specimens. Specimens (geometry in Figure 11) were tested on an HDT105B electro-hydraulic servo fatigue testing machine (WanCe Testing Equipment Co., Ltd., Jinan, Shandong Province, China) at room temperature with a loading rate of 2 mm/min. Three repeated experiments were performed, and the results were highly consistent. The average results are yield strength 505 Mpa and ultimate tensile strength 864 Mpa. The stress–strain curve of one representative experiment is shown in Figure 12a,b.

A total of ten specimens were tested, including four uncorroded specimens and three specimens each pre-corroded for 15 and 30 days, respectively. The accelerated corrosion method adopted in this section was identical to that described in the previous section (Section 2). All specimens were subjected to constant-amplitude cyclic loading. The tensile fatigue tests were performed using the HDT105B electro-hydraulic servo fatigue testing machine (China WanCe Group), with a capacity range of ±100 kN.

The selection of fatigue testing conditions was based on material properties, corrosion damage characteristics, and engineering application background. (1) Stress amplitude range (290–449.28 MPa): Derived from the measured yield strength of the virgin material (505 MPa), covering 57–89% of yield strength to span crack initiation and stable propagation stages. This avoids excessively long test durations at low loads or immediate fracture at high loads. (2) Loading ratios (R = 0.03/0.2): R = 0.2 was used as the standard reference for intrinsic fatigue properties, and R = 0.03 simulated typical marine service conditions (low mean stress for ship propellers) to evaluate corrosion-induced fatigue behavior under realistic operating conditions. (3) Loading frequency (5 Hz/30 Hz): Preliminary tests were conducted at 5 Hz, but subsequent fatigue tests were uniformly adjusted to 30 Hz to improve experimental efficiency and reduce time costs.

The test conditions and results are shown in Table 5.

All references to ‘stress’ in this paper specifically refer to ‘axial normal stress’, and ‘stress’ will be used as an abbreviation for ‘axial normal stress’ hereafter.

To account for mean stress effects, Goodman diagram correction was applied to R = 0.03 data:

$$\sigma_{a}^{\prime }=\sigma_{a}\cdot \frac{1-R}{1-R_{0}}$$

where $\sigma_{a}^{\prime }$ represents corrected stress amplitude (equivalent to R=0.2), $\sigma_{a}$ represents measured stress amplitude, and $R_{0}=0.2$ represents reference ratio. The corrected data were used for comparison and plotting of the S–N curves.

### 3.2. S–N Curves Without Corrosion

Currently, there are very limited data on the fatigue strength of nickel–aluminum bronze, with only the Germanischer Lloyd (GL) classification society and the Transportation Safety Administration providing S–N curves for bronze propellers operating in ice conditions [28,29]. In 1975, some scientists validated the officially published S–N curves for nickel–aluminum bronze materials [30,31,32], where fatigue test specimens were cut from actual propeller blades and tested at frequencies ranging from 28–33 Hz and 50 Hz to 160 Hz.

**Figure 13:**
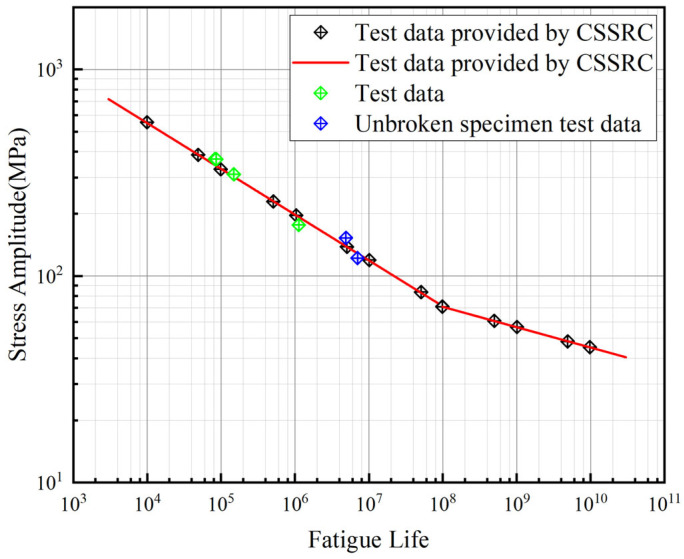
S–N curve of nickel–aluminum bronze.

Due to variations in chemical composition and fatigue loading conditions, the S–N curves of nickel–aluminum bronze exhibit significant differences. To establish the S–N curve for the specific nickel–aluminum bronze ZCuAl_8_Mn_13_Fe_3_Ni_2_ used in this project, experimental data from corrosion-free fatigue tests (including four specimens and additional fatigue life data at various stress levels provided by CSSRC [33]) were fitted to derive the material’s characteristic S–N curve, as shown in Figure 13.

lgS=A−BlgN

where *A* = 3.204, *B* = 0.172 (before the knee); *A* = 2.639, *B* = 0.099 (after the knee).

The derived curve demonstrates close agreement with the experimental validation results of the officially published S–N curves for nickel–aluminum bronze materials reported by scientists in 1975 [30,31,32].

### 3.3. Fatigue Life After Accelerated Corrosion

**Figure 14:**
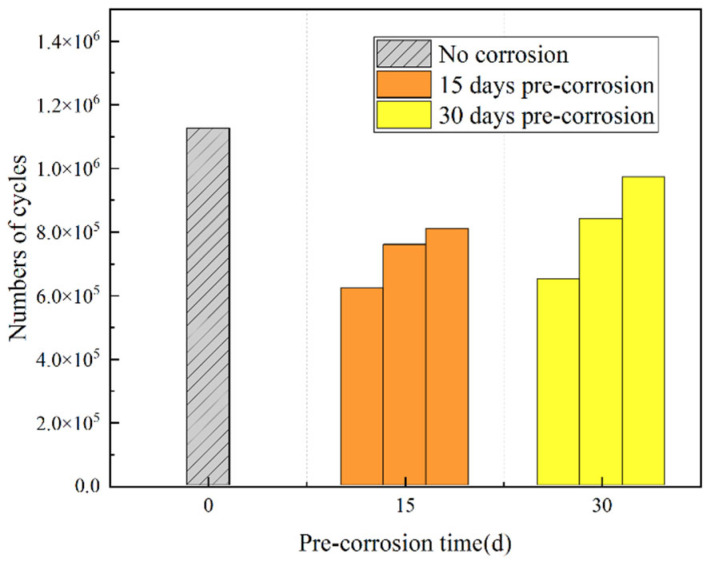
Fatigue life of specimens.

Figure 14 shows the mean fatigue life of uncorroded specimens at identical stress levels and the lifetime comparison between uncorroded specimens and those with 15d/30d accelerated corrosion exposure.

As shown in Table 5 and Figure 14, the effects of pre-corrosion treatment vary for each nickel–aluminum bronze specimen, and the fatigue life demonstrates significant dispersion among different specimens. Both 15d and 30d pre-corrosion treatments reduce the fatigue lives of nickel–aluminum bronze specimens, with a maximum reduction of 44.67%. The average fatigue life of the three 15d pre-corroded specimens (7.30 × 10^5^) is lower than that of the three 30d pre-corroded specimens (8.20 × 10^5^). The lower fatigue life of the 15d pre-corroded specimens may be attributed to the following reasons: (1) A shorter pre-corrosion period (e.g., 15d) may not allow the formation of a stable corrosion layer on the specimen surface, leading to irregular and non-uniform corrosion pit distribution. This non-uniform distribution can induce stress concentration, increasing the likelihood of fatigue crack initiation. In comparison, 30d pre-corrosion may promote the formation of a more uniform or thicker corrosion layer, which could mitigate stress concentration and extend fatigue life. (2) Corrosion affects the surface morphology and structural integrity of the metal specimens, leading to reductions in yield strength and ultimate tensile strength [34,35], thereby decreasing their fatigue resistance. In the initial stage (e.g., 15d), shallow and dense corrosion pits induce competitive initiation of multiple cracks, leading to a significant decrease in material strength and thus a drastic reduction in fatigue life. In the later stage (e.g., 30d), corrosion promotes the growth of deep and isolated pits through preferential pit development, accompanied by the formation of a dense oxide film. Although the material strength continues to decline, the oxide film inhibits crack initiation, reducing the propagation rate of the single main crack and ultimately resulting in extended fatigue life.

### 3.4. Fatigue Fracture Surface Analysis of Pre-Corroded Specimens

To investigate the fatigue crack initiation sites of nickel–aluminum bronze specimens subjected to varying durations of pre-corrosion treatment, the fatigue fracture surfaces of three specimens were meticulously examined using a GeminiSEM 500 scanning electron microscope (ZEISS Group, Oberkochen, Germany).

**Figure 15:**
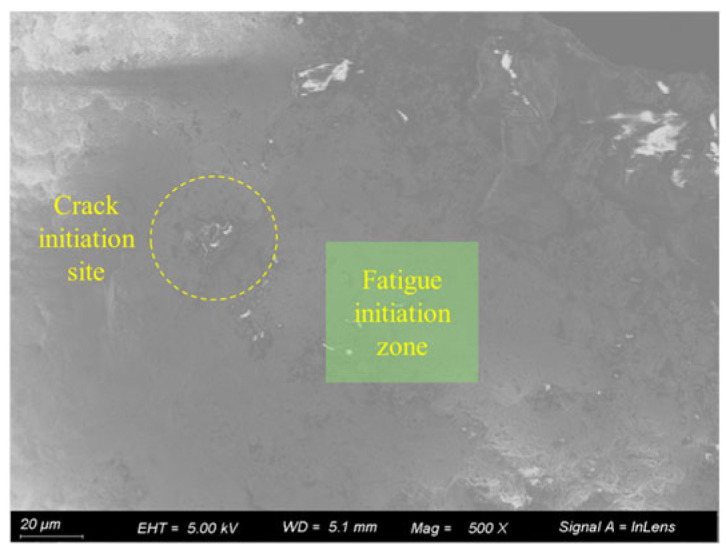
Fracture surface of uncorroded specimen (20 μm).

Figure 15 shows the fatigue fracture surface of an uncorroded specimen. As observed in the figure, fatigue cracks in the uncorroded specimen initiated at internal particle inclusions near the specimen surface. These cracks propagated progressively under cyclic loading until the final fracture occurred.

**Figure 16:**
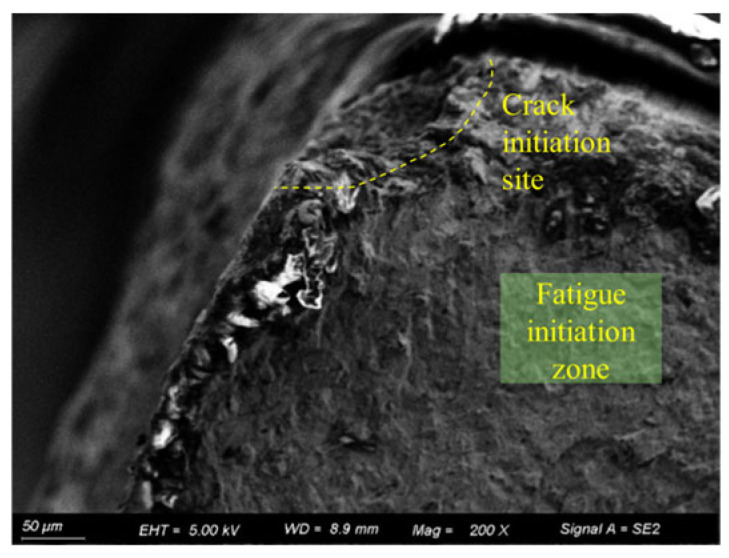
Fracture surface of 15d pre-corroded specimen (50 μm).

Figure 16 shows the fatigue fracture surface of the 15d pre-corroded specimen. As observed in the figure, the crack initiation source of the 15d pre-corroded specimen is located at a corrosion pit in the corner of the specimen cross-section. The pit is overall semi-elliptical with an aspect ratio of approximately 1:2.

**Figure 17:**
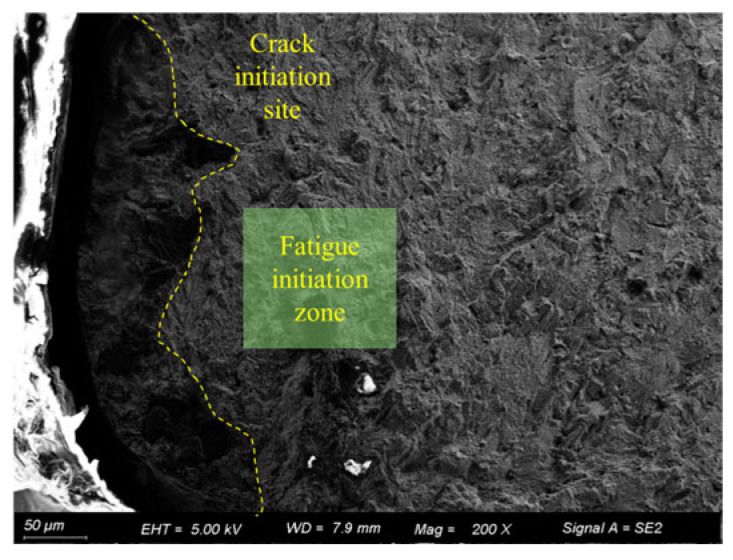
Fracture surface of 30d pre-corroded specimen (50 μm).

Figure 17 shows the fatigue fracture surface of the 30d pre-corroded specimen. As observed in the figure, the crack initiation source of the 30d pre-corroded specimen is also located at a corrosion pit in the corner of the specimen cross-section. However, this pit is formed by the connection of multiple pits of varying sizes, exhibiting an irregular shape.

The analysis indicates that corrosion leads to the formation of unevenly distributed corrosion pits with varying sizes on the specimen surface, resulting in progressive deterioration of surface roughness with prolonged exposure time. Due to stress concentration at these corrosion pits—where the stress intensity factor exceeds that at internal inclusions—fatigue cracks preferentially initiate at the pit sites.

## 4. Corrosion Fatigue Numerical Simulations

The study of metal corrosion fatigue behavior presents significant complexity due to the coupled electrochemical–mechanical interactions involved. Unlike pure mechanical fatigue or static corrosion, the combined action of cyclic loading and corrosive environments creates unique failure mechanisms. Specifically, applied loads increase the metal’s chemical potential, lowering the activation energy barrier for anodic dissolution and altering the equilibrium potential of metal oxidation, while mechanical deformation-induced dislocation multiplication and surface defects modify the cathodic exchange current density. To address this, the present research employs COMSOL Multiphysics^®^ software (Version 6.3, COMSOL Inc., Stockholm, Sweden) that integrates fatigue analysis with secondary current distribution modeling, combining nickel–aluminum bronze’s electrochemical corrosion behavior with fatigue performance to predict its corrosion fatigue life. This approach quantitatively accounts for the synergistic effects between mechanical damage and electrochemical corrosion processes.

### 4.1. Theory of Corrosion Fatigue

#### 4.1.1. Theory of Corrosion

Marine structures constructed from nickel–aluminum bronze materials form a short-circuited galvanic cell system when exposed to seawater during service. The seawater environment typically serves as an oxygen-rich, weakly alkaline electrolyte medium. The principal anodic and cathodic reactions of nickel–aluminum bronze in seawater are as follows:

(1) $$\left\{\begin{array}{l} Cu\to Cu^{2+}+2e^{-}\\ O_{2}+2H_{2}O+4e^{-}\to 4{\mathrm{OH}}^{-} \end{array}\right.$$

For the two-electrode reactions outlined in Equation (1), the absolute electrode potentials cannot be measured directly. However, by utilizing the stable electrode potential of a reference electrode, the relative potential between the reference electrode and the working electrode during the reactions—known as the equilibrium potential—can be determined. The equilibrium potentials for these reactions can then be calculated using the Nernst equation [36]:

(2) $$\left\{\begin{array}{l} E_{e(C{u/\mathrm{Cu}}^{2+})}=E_{{\mathrm{Cu}/\mathrm{Cu}}^{2+}}^{0}+\frac{RT}{2F}\ln a_{Cu^{2+}}\\ E_{e({\mathrm{OH}}^{-}{/O}_{2})}=E_{{\mathrm{OH}}^{-}{/O}_{2}}^{0}+\frac{RT}{4F}\ln\frac{p_{O_{2}}}{a_{{\mathrm{OH}}^{-}}^{4}} \end{array}\right.$$

where $E_{{\mathrm{Cu}/\mathrm{Cu}}^{2+}}^{0}$ represents the standard electrode potential of the anodic reaction, defined as the equilibrium electrode potential under conditions of a metal activity of 1, a temperature of 298.15 K, and a pressure of 1 atm; $E_{{\mathrm{OH}}^{-}{/O}_{2}}^{0}$ represents the standard electrode potential of the cathodic reaction, defined as the equilibrium electrode potential under conditions of a fugacity of 1, a temperature of 298.15 K, and a pressure of 1 atm; *R* represents the ideal gas constant; *T* represents the thermodynamic temperature in Kelvin; $a_{Cu^{2+}}$ represents the activity of Cu^2+^ ions in the electrolyte solution, which in dilute solutions can be approximated by the molar concentration of the corresponding ions with units of mol/cm^3^; $a_{{\mathrm{OH}}^{-}}$ represents the activity of OH^−^ ions in the electrolyte solution with units of mol/cm^3^; $P_{O_{2}}$ represents the partial pressure of oxygen in the electrolyte solution, with units of atm.

The relationship between electrode current density and overpotential is governed by the Butler–Volmer equation [37,38]. In seawater, nickel–aluminum bronze can be considered as an isolated electrode. Assuming the potential of nickel–aluminum bronze in seawater is E, the absolute values of the anodic and cathodic net currents for nickel–aluminum bronze can be derived from the Butler–Volmer equation when the two electrode reactions in Equation (1) occur on the electrode surface, as follows:

(3) $$\left\{\begin{array}{l} I_{a}=I_{0,a}\left\{\left.\exp\left(\frac{2F\alpha_{a}(E-E_{e,a})}{RT}\right)-\exp\left[-\frac{2F(1-\alpha_{a})(E-E_{e,a})}{RT}\right]\right\}\right.\\ \left|I_{c}\right|=I_{0,c}\left\{\left.\exp\left(-\frac{4F(1-\alpha_{c})(E-E_{e,c})}{RT}\right)-\exp\left[\frac{4F\alpha_{c}(E-E_{e,c})}{RT}\right]\right\}\right. \end{array}\right.$$

In Equation (3), $\alpha_{a}$ and $\alpha_{c}$ represent the transfer coefficients of the anodic and cathodic reactions, respectively; $I_{0.a}$ and $I_{0.c}$ represent the exchange current densities associated with the anodic and cathodic reactions, respectively; $E_{e.a}$ and $E_{e.c}$ represent the equilibrium potentials of the anodic and cathodic reactions, respectively, as determined by Equation (2).

Under self-corrosion conditions in seawater, the absolute values of the current densities for the anodic and cathodic reactions of nickel–aluminum bronze are equal, and the external current is effectively zero. At this point, the potential of nickel–aluminum bronze corresponds to its corrosion potential, represented by $E_{corr}$. Since the corrosion potential of nickel–aluminum bronze significantly deviates from the equilibrium potentials of the electrode reactions, the reverse reaction terms in both the anodic and cathodic reactions can be neglected. As a result, Equation (3) can be simplified to the Tafel equation [39], where the constant terms are expressed in terms of the natural logarithm of the Tafel slopes $\beta_{a}$ and $\beta_{c}$. The simplified result is given in Equation (4).

(4) $$\left\{\begin{array}{l} I_{a}=I_{0,a}\exp\left(\frac{\left(E-E_{e,a}\right)}{\beta_{a}}\right)\\ \left|I_{c}\right|=I_{0,c}\exp\left(-\frac{\left(E-E_{e,c}\right)}{\beta_{c}}\right) \end{array}\right.$$

When the potential of the nickel–aluminum bronze specimen equals its corrosion potential, the current densities of both the anodic and cathodic reactions are equal and equivalent to the corrosion current density $I_{corr}$. The relationship among these three parameters is described by Equation (5):

(5) $$I_{corr}=I_{0,a}\exp\left(\frac{\left(E_{corr}-E_{e,a}\right)}{\beta_{a}}\right)=I_{0,c}\exp\left(-\frac{\left(E_{corr}-E_{e,c}\right)}{\beta_{c}}\right)$$

By combining Equations (4) and (5) and eliminating the equilibrium potentials and exchange current densities associated with the anodic and cathodic reactions in Equation (4), the current density on the external surface of nickel–aluminum bronze is derived. The relationship between the current density and the polarization value is expressed in Equation (6) as follows:

(6) $$\left\{\begin{array}{l} I=I_{a}-\left|I_{c}\right|=I_{corr}\left[\exp\left(\frac{\Delta E}{\beta_{a}}\right)-\exp\left(-\frac{\Delta E}{\beta_{c}}\right)\right]\\ \Delta E=E-E_{corr} \end{array}\right.$$

In Equation (6), the equilibrium potentials of the anodic and cathodic reactions are replaced by the corrosion potential, establishing the relationship between the polarization value ΔE and the external current I. This equation describes the polarization curve of nickel–aluminum bronze under seawater environments.

#### 4.1.2. Corrosion–Fatigue Synergy

The applied load and corrosion factors interact with each other, exhibiting a coupled effect where structural corrosion leads to changes in dimensions (e.g., thickness reduction), thereby altering stress distribution, while mechanical loading modifies corrosion behavior. According to Gutmann’s research, mechanical loading elevates the metal’s chemical potential, affecting the equilibrium potential of anodic reactions [40]. Simultaneously, mechanical deformation generates surface microcracks and slip steps, which alter the cathodic exchange current density. The corrosion model in this study is developed based on an electrochemical framework, enabling the coupling between mechanical effects and secondary current distribution by modifying the anodic equilibrium potential and cathodic exchange current density of the structure. When subjected to loading, the following load-dependent terms can be incorporated into the anodic equilibrium potential and cathodic exchange current density of the structure:

(7) $$\Delta E_{e\left(Cu/Cu^{2+}\right)}=-\frac{V\Delta P}{2F}-\frac{RT}{2F}\ln\left(\frac{0.45\alpha }{N_{0}}\varepsilon +1\right)$$

(8) $${\left(i_{0,c}\right)}_{stress}=i_{0,c}\cdot 10^{\frac{-\sigma V}{6Fb_{c}}}$$

where *V* represents the molar volume of material; ΔP represents the spherical part of the stress tensor. It can be taken as the maximum principal stress under volumetric stress; *T* represents the thermodynamic temperature; α represents the coefficient, which is taken as 1.67 × 10^11^ cm^−2^ [41,42]; ε represents the small plastic deformation; $N_{0}$ represents the initial material density; σ represents the Von mises stress.

Structural materials continue to obey the Butler–Volmer equation even when subjected to mechanical loading.

(9) $$\left\{\begin{array}{l} I_{a}=I_{0,a}\exp\left(\frac{E-\left(E_{e,a}+\Delta E_{e\left(Cu/Cu^{2+}\right)}\right)}{\beta_{a}}\right)\\ \left|I_{c}\right|={\left(I_{0,c}\right)}_{stress}\exp\left(-\frac{\left(E-E_{e,c}\right)}{\beta_{c}}\right) \end{array}\right.$$

where, $I_{0,a}$ and $I_{0,c}$ represent the exchange current densities for the anodic and cathodic reactions, respectively; $E_{e,a}$ and $E_{e,c}$ represent the equilibrium potentials for the anodic and cathodic reactions, respectively.

The boundary condition for the model is established as follows:

(10) $$I=I_{corr}\left[\exp\left(\frac{E-E_{corr}-\Delta E_{e\left(Cu/Cu^{2+}\right)}}{\beta_{a}}\right)-10^{\frac{-\sigma V}{6Fb_{c}}}\cdot \exp\left(-\frac{\left(E-E_{corr}\right)}{\beta_{a}}\right)\right]$$

The equation characterizes the electrode kinetic behavior under mechanical loading, incorporating the influence of mechanical effects on corrosion processes. By introducing the aforementioned load-dependent terms into the boundary conditions of the corrosion model, the mechanical coupling from the solid mechanics module to the electrochemical module can be achieved. Furthermore, within the corrosion model itself, a bidirectional coupling relationship exists between the electrochemical module and the deformation geometry module. If the deformation geometry module and electrochemical module are collectively termed as the corrosion module, this enables coupled bidirectional interactions between the corrosion module and the solid mechanics module.

### 4.2. Simulation Models and Parameters of Corrosion Fatigue

#### 4.2.1. Parameter Settings

During the numerical simulation process, the key material parameter inputs include specimen dimensions, material parameters, electrochemical parameters, and boundary loading conditions. The specimen dimensions correspond to the fatigue test specimen shown in Figure 11 of Section 3.1. The key material parameters input include density, molar mass, molar volume, Young’s modulus, and Poisson’s ratio. The corrosion solution is the accelerated corrosion condition with a high corrosion rate as described in Section 2.1. The electrochemical parameters of nickel–aluminum bronze in this corrosive medium, including equilibrium potential, self-corrosion current density, cathodic Tafel slope, and anodic Tafel slope, were obtained from potentiodynamic polarization tests (Figure 4 in Section 2.1 shows the polarization curve results). The boundary loading conditions were configured according to the fatigue loading parameters for corroded components shown in Table 5. The detailed parameter settings are shown in Table 6.

#### 4.2.2. Mesh Generation and Governing Equations

**Figure 18:**
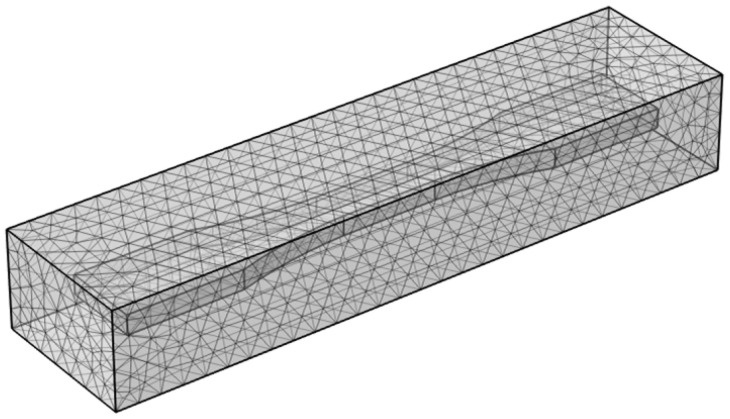
Ultra-fine mesh model for electrolyte and electrode domains.

The corrosion simulation model involves meshing two computational domains: the electrolyte domain (representing the accelerated corrosion environment) and the electrode domain (representing the specimen). To ensure convergence of corrosion calculations, both domains are discretized using ultra-fine free tetrahedral meshes. During the corrosion process, mesh deformation occurs. To prevent severe mesh quality degradation that could compromise simulation accuracy, an adaptive remeshing algorithm is implemented to automatically regenerate the mesh when distortion exceeds critical thresholds. The mesh configuration is shown in Figure 18.

According to the law of mass conservation and neglecting source terms from electrochemical reactions in the governing equations, the concentration and flux of substance a in the electrolyte satisfy Equation (11):

(11) $$\frac{\partial c_{i}}{\partial t}+\nabla N_{i}=0$$

where $c_{a}$ represents the concentration of substance a, and $N_{a}$ represents the flux of substance a.

Considering the diffusion, electromigration, and convective effects in the electrolyte, the flux $N_{a}$, and current density $i_{b}$ can be described by the Nernst–Planck equation [43,44]:

(12) $$\left\{\begin{array}{l} N_{a}=-D_{a}\nabla c_{a}-z_{a}u_{a}Fc_{a}\nabla \varphi_{b}+c_{a}V\\ i_{b}=F\sum_{a}z_{a}N_{a} \end{array}\right.$$

where $D_{a}$ represents the diffusion coefficient of the substance a; F represents Faraday’s constant; $u_{a}$ represents the ion mobility; $z_{a}$ represents the charge number of the substance; $\phi_{b}$ represents the potential of the electrolyte; and V represents the velocity of the electrolyte.

In the Secondary Current Distribution module of COMSOL’s electrochemistry interface, the software accounts for the influence of electrode kinetics and solution resistance while adopting two key assumptions: (1) Electroneutrality of the electrolyte: The electrolyte solution is assumed to be electrically neutral, and convective transport effects are neglected; (2) uniform chemical composition: Variations in electrolyte composition are considered negligible, with the overall distribution treated as homogeneous.

Considering the two assumptions of the Secondary Current Distribution module, the potential distribution in the electrolyte domain can be simplified to an Ohm’s Law equation [45]:

(13) $$\left\{\begin{array}{l} i_{b}=-\sigma_{b}\nabla \phi_{b}\\ \sigma_{b}=F^{2}\sum_{a}z_{a}^{2}u_{a}c_{a} \end{array}\right.$$

Thus, the electrolyte potential $\phi_{b}$ and electrode potential $\phi_{c}$ satisfy the following potential governing equations:

(14) $$\left\{\begin{array}{l} i_{b}=-\sigma_{b}\nabla \phi_{b}\\ i_{c}=-\sigma_{c}\nabla \phi_{c}\\ \nabla \cdot i_{b}=Q_{b}\\ \nabla \cdot i_{c}=Q_{c} \end{array}\right.$$

The boundaries of the electrolyte domain satisfy the equation:

(15) $$\nabla_{n}\phi_{b}=0$$

#### 4.2.3. Process of Corrosion Fatigue Numerical Simulation

This study employs a numerical simulation approach to conduct a coupled analysis of mechanical loading and corrosion, integrating COMSOL’s Solid Mechanics module with fatigue analysis. The fatigue analysis procedure and methodology based on MATLAB co-simulation are as follows: (1) Utilizing API interfaces, MATLAB programs read and store the mesh model at specified corrosion time steps from COMSOL; (2) the stored mesh models are recalled into COMSOL(Version 6.3) via MATLAB(Version 2021a) programs for stress–strain computation; and (3) MATLAB reads and stores stress values of all model elements, performs hot-spot stress interpolation calculations, and ultimately conducts fatigue damage assessment at critical locations based on S–N curves.

**Figure 19:**
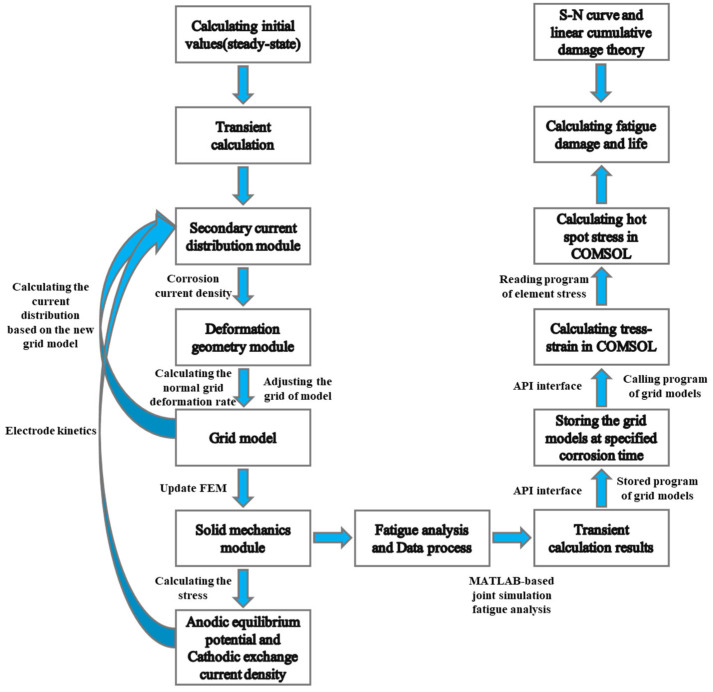
Flowchart of numerical simulation for corrosion fatigue analysis.

The workflow and methodology for the aforementioned corrosion–fatigue simulation analysis are shown in Figure 19:

## 5. Fatigue Life Prediction in Seawater Corrosion Environments

### 5.1. Model Validation

To validate the accuracy of the corrosion–fatigue numerical simulation in Section 3, this section first calculates the corrosion acceleration factor for nickel–aluminum bronze specimens in the accelerated corrosion environment and compares it with the corrosion rates obtained in Section 2.2. Additionally, numerical simulations were performed for four corrosion–fatigue loading conditions, with the first three cases compared against the experimental corrosion–fatigue test results from Section 2.

#### 5.1.1. Corrosion Rate

**Figure 20:**
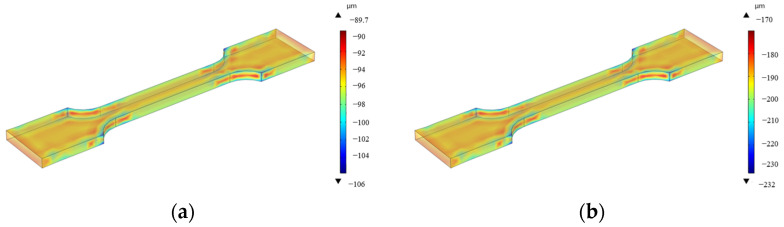
Corrosion thickness distribution of specimens in accelerated corrosion environment: (**a**) 15 days pre-corrosion, (**b**) 30 days pre-corrosion.

The simulated corrosion thickness after 15d and 30d of accelerated corrosion is shown in Figure 20, from which the corrosion rate can be calculated. A comparison between the numerically calculated corrosion rates and experimental results from Section 2.2 is shown in Table 7.

Through comparative analysis, it can be concluded that the numerical calculation results closely align with experimental data. The maximum observed deviation of 1.06% occurred under 30d pre-corrosion conditions, with all errors remaining within acceptable thresholds. This demonstrates the reliability of the numerical results and confirms the correctness and effectiveness of the corrosion simulation model.

#### 5.1.2. Corrosion Fatigue Life Prediction

The corrosion fatigue life of nickel–aluminum bronze specimens was numerically calculated under four conditions (no corrosion, 15d pre-corrosion, 30d pre-corrosion, and coupled corrosion–fatigue interaction) using the methodology and parameters defined in Section 3, with identical fatigue loading (stress range: 10–300 MPa, amplitude: 145 MPa, frequency: 30 Hz) applied across all cases. The computational results and corresponding experimental data from Section 2 for the first three conditions are compared in Table 8.

Based on the data analysis from Table 8, it can be concluded that

1. For the no corrosion condition, the predicted life showed a 4.90% deviation from experiments, within the 10% acceptable threshold.
2. For the pre-corrosion condition (15d/30d exposure), prediction errors reached 15.71% and 11.94%, respectively, all below the 20% margin.
3. Comparison between the no corrosion and coupled corrosion–fatigue conditions revealed an 11.64% life reduction, demonstrating the coupled corrosion–fatigue interaction mechanism.

**Figure 21:**
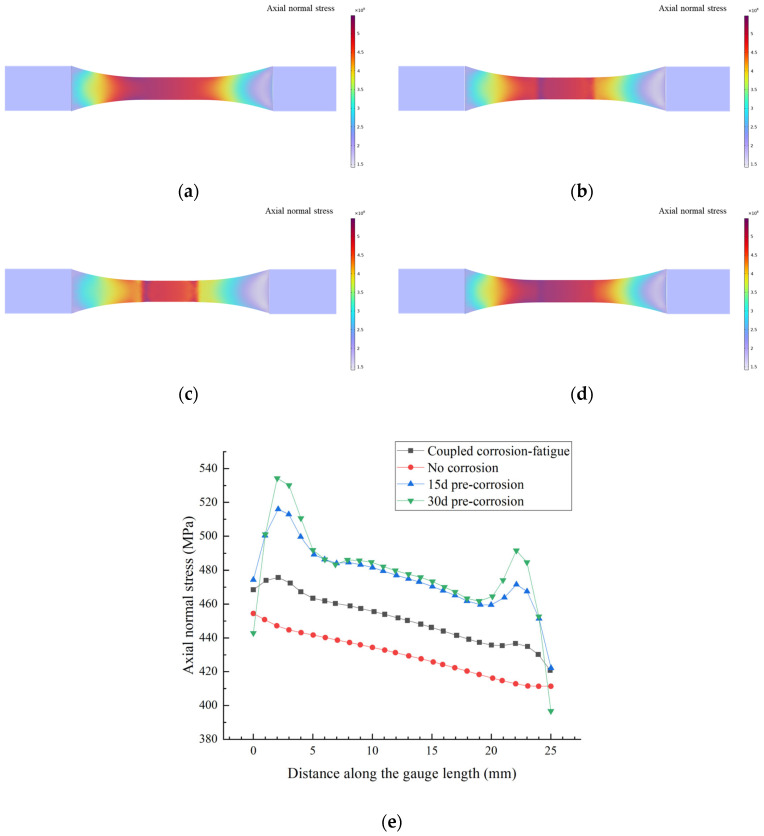
Stress distribution at failure under different corrosion–fatigue conditions: (**a**) No corrosion, (**b**) 15d pre-corrosion, (**c**) 30d pre-corrosion, (**d**) Coupled corrosion–fatigue, (**e**) Stress variation in the test sections of the specimens.

When the damage accumulation reaches unity (*D* = 1), the stress contour at specimen fatigue failure is shown in Figure 21.

As shown in Figure 21a, the stress distribution of the uncorroded specimen exhibits a relatively regular pattern, which is correlated with the specimen’s geometric shape, and the maximum stress occurs at the cross-section transition zone. In Figure 21b,c, the pre-corroded specimens experience thickness reduction and cross-sectional changes, leading to a more concentrated stress distribution, where the maximum stress increases with prolonged pre-corrosion time. Figure 21d shows that the stress distribution of the corrosion–fatigue coupled specimen is similar to that of the pre-corroded specimen, with corrosion-induced stress concentration. Under constant applied load, corrosion causes a reduction in the cross-sectional area, resulting in increased stress, and the magnitude of stress amplification becomes more pronounced with extended corrosion exposure time.

#### 5.1.3. S–N Curve Modification After Accelerated Corrosion

**Figure 22:**
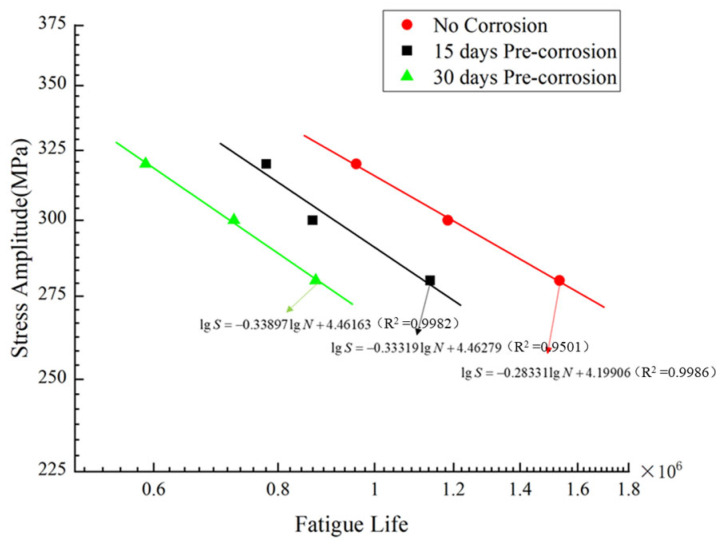
Modified S–N curve.

Seawater corrosion environments accelerate the fatigue failure process of materials. It is therefore essential to modify the S–N curves for the nickel–aluminum bronze material after corrosion exposure in this study. Based on numerical simulation methods, this research calculated the stress–fatigue life relationships for specimens subjected to 15d and 30d pre-corrosion. The modified S–N curves for both the no-corrosion and pre-corrosion conditions are shown in Figure 22.

**Figure 23:**
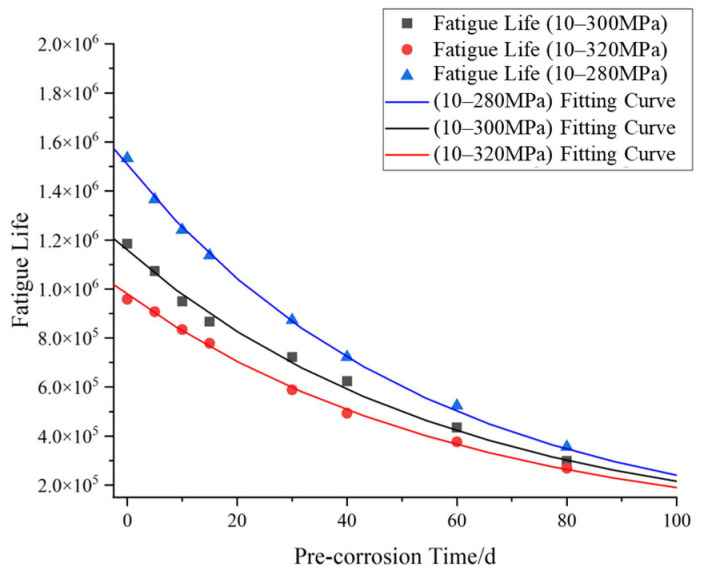
Fatigue life vs. corrosion time relationship.

The modified S–N curves were incorporated into COMSOL to calculate the fatigue life of specimens under three stress ranges (10–300 MPa, 10–320 MPa, and 10–280 MPa) after 0, 5, 10, 15, and 30 days of pre-corrosion exposure. Based on the computational results, the fatigue life of specimens under different stress levels and pre-corrosion durations was plotted, with corresponding fitting curves generated as shown in Figure 23.

As shown in Figure 23, under the same stress range, the fatigue life of the specimen decreases non-linearly with the prolongation of pre-corrosion time, indicating that pre-corrosion has a negative impact on the fatigue life of the material, and this impact increases with the prolongation of pre-corrosion time. Under the same pre-corrosion time, the higher the stress level, the lower the fatigue life of the specimen, indicating that the higher the stress level, the more sensitive the material is to pre-corrosion, and the faster the fatigue life decreases.

## 6. Conclusions

This paper focuses on the ZCuAl_8_Mn_13_Fe_3_Ni_2_ nickel–aluminum bronze. Through electro-chemical tests and corrosion weight loss tests, the corrosion influencing factors of nickel–aluminum bronze are mastered. A corrosion acceleration test method for the marine full immersion zone is proposed. Combined with the pre-corrosion fatigue test in the corrosion acceleration solution environment, the corrosion damage characteristics of nickel–aluminum bronze and its influence on fatigue performance are analyzed. Its pre-corrosion fatigue damage mechanism is revealed. The test results are compared with the numerical simulation method based on COMSOL and MATLAB, which shows that it can accurately predict the pre-corrosion fatigue life of materials. Finally, the S–N curves of the material under different pre-corrosion times are established. The main conclusions are as follows:

1. Elevating the temperature of the corrosion solution and decreasing the pH value have a significant impact on enhancing the corrosion rate of the ZCuAl_8_Mn_13_Fe_3_Ni_2_ nickel–aluminum bronze. Using the test solution of 0.6 mol/L NaCl + 0.1 mol/L H_3_PO_4_-NaH_2_PO_4 _buffer solution + 1.0 mol/L H_2_O_2_ + 0.1 mL/500 mL concentrated hydrochloric acid for the acceleration test has good corrosion acceleration. The corrosion weight loss of the material in the corrosion acceleration environment for 15 days is equivalent to that in the full immersion zone of natural seawater for 56 months, and the corrosion acceleration multiple is approximately 110.83 times.
2. In the environment of the corrosion acceleration solution, due to the stress concentration caused by the corrosion pits on the material surface, the fatigue life of nickel–aluminum bronze is significantly reduced. The average fatigue life of the pre-corroded specimens for 15–30 days is approximately 27.18–34.14% of that of the original specimens. With the generation of a large amount of stable corrosion products in the later stage of the test, the densification and protective ability of the rust layer are significantly improved. Therefore, with the increase of the corrosion cycle, the attenuation trend of the fatigue life gradually slows down.
3. The combined simulation method of corrosion fatigue based on COMSOL and MATLAB proposed in this paper can accurately simulate the influence of corrosion damage on the fatigue performance of materials. The average deviation of corrosion damage simulation based on corrosion weight loss is 0.61%, and the average error of the fatigue prediction of pre-corroded specimens is 13.82%.
4. Taking into account the impact of seawater corrosion on material performance, the S–N curves of nickel–aluminum bronze in the corrosive environment were modified. Pre-corrosion has a negative impact on the fatigue performance of specimens. With the increase of pre-corrosion time (from 0 days to 15 days and then to 30 days), the fatigue strength of specimens decreases, and fatigue failure is more likely to occur.

## Figures and Tables

**Table 1:** Chemical composition and mass fraction of ZCuAl_8_Mn_13_Fe_3_Ni_2_ nickel–aluminum bronze.

Composition	Al	Mn	Fe	Ni	Zn	Pb	Si	C	Cu
Mass Fraction %	8.0	13.0	3.0	2.0	<0.3	<0.02	<0.15	<0.1	Balance

**Table 2:** Accelerated corrosion testing conditions.

Batch Number	Temperature °C	PH Value	Environment	Self-Corrosion Current Density, μA·cm^−2^	Self-Corrosion Potential, V
1	25	7	Artificial Seawater	1.413	−0.250
2	25	7	Natural Seawater	1.349	−0.212
3	50	1	0.1 mol/L HCl	7.943	−0.309
4	50	0.5	0.316 mol/L HCl	11.480	−0.288
5	60	1	0.1 mol/L HCl	9.120	−0.312
6	60	0.5	0.316 mol/L HCl	12.288	−0.287
7	25	--	A solution containing 0.6 mol/L NaCl, 0.1 mol/L H_3_PO_4_, 0.1 mol/L NaH_2_PO_4_ as a buffer, and 1 mol/L H_2_O_2_, with hydrochloric acid added at a volume fraction of 0.1 mL per 500 mL [24]	288.403	0

**Table 3:** Chemical Composition of Artificial Seawater.

Component	NaCl	MgCl_2_	Na_2_SO_3_	CaCl_2_	KCl
Content, g/L	24.53	5.20	4.09	1.16	0.695
Component	NaHCO_3_	KBr	H_3_BO_3_	SrCl_2_	NaF
Content, g/L	0.201	0.101	0.027	0.025	0.003

**Table 4:** Surface roughness parameters of corroded specimens.

Parameter	*S*_a_/μm	*S*_ku_	*S*_p_/μm	*S*_q_/μm	*S*_sk_	*S*_v_/μm	*S_z_*/μm
15d	16.930	13.562	319.717	33.666	−2.481	−414.931	734.648
30d	32.930	6.354	136.087	45.181	−1.257	−256.204	392.291

**Table 5:** Fatigue load level and fatigue life.

Operating Conditions	Specimen Number	Loading Frequency Hz	Loading Range (Axial Normal Stress) MPa	Stress Amplitude MPa	Fatigue Life	Percentage Reduction %
No corrosion	NC-1	5	112.32–561.6	224.64	82,655	--
	NC-2	5	112.32–561.6	224.64	86,100	--
	NC-3	30	48–480	216	148,842	--
	NC-4	30	10–300	145	11.26 × 10^5^	--
Unbroken	NC-5	30	13–224	105.5	--	--
	NC-6	30	10–267	128.5	--	--
Pre- corrosion for 15 days	PC15-1	30	10–300	145	6.23 × 10^5^	44.67
	PC15-2	30	10–300	145	7.59 × 10^5^	32.59
	PC15-3	30	10–300	145	8.09 × 10^5^	25.15
Pre- corrosion for 30 days	PC30-1	30	10–300	145	6.48 × 10^5^	42.45
	PC30-2	30	10–300	145	8.40 × 10^5^	25.4
	PC30-3	30	10–300	145	9.72 × 10^5^	13.68

**Table 6:** Parameter list.

Parameter	Value	Unit
Density	8.679	g/m^3^
Molar mass	62.14	g/mol
Molar volume	7.09	cm^3^/mol
Young’s modulus	123	GPa
Poisson’s ratio	0.33	/
Equilibrium potential	0	V
Self-corrosion current density	288.403	μA·cm^−2^
Cathodic Tafel slope	124.45	mV
Anodic Tafel slope	−85.99	mV
Fatigue loading	10–300	MPa

**Table 7:** Comparison between numerical calculations and experimental results of corrosion rate.

Specimen	Numerically Computed Corrosion Rate	Experimentally Measured Corrosion Rate	Relative Error
15d	6.28 μm/d	6.29 μm/d	0.16%
30d	6.44 μm/d	6.51 μm/d	1.06%

**Table 8:** Predicted fatigue life of corroded specimens.

Corrosion–Fatigue Conditions	Predicted Fatigue Life	Mean Experimental Fatigue Life	Relative Error
no corrosion	1,184,400	1,126,344	4.90%
15d pre-corrosion	866,600	730,471	15.71%
30d pre-corrosion	722,300	820,208	11.94%
Coupled corrosion–fatigue	1,073,000		

## Data Availability

The original contributions presented in this study are included in the article. Further inquiries can be directed to the corresponding author(s).
